# A critical review speculating on the protective efficacies of autogenous *Streptococcus suis* bacterins as used in Europe

**DOI:** 10.1186/s40813-020-00150-6

**Published:** 2020-05-06

**Authors:** Karoline Rieckmann, Sophia-Mareike Pendzialek, Thomas Vahlenkamp, Christoph G. Baums

**Affiliations:** 1 Institute of Bacteriology and Mycology, Centre for Infectious Diseases, Faculty of Veterinary Medicine, University Leipzig, An den Tierkliniken 29, 04103 Leipzig, Germany; 2 Institute of Virology, Centre for Infectious Diseases, Faculty of Veterinary Medicine, University Leipzig, Leipzig, Germany

**Keywords:** Maternal immunity, PRRSV, Influenza, MRP, EF, Meningitis, Adjuvant, Opsonizing antibodies

## Abstract

**Background:**

*Streptococcus (S.) suis* is a major porcine pathogen causing high morbidity worldwide. This includes well-managed herds with high hygiene standards. In Europe, no licensed vaccine is available. As practitioners are obliged to reduce the use of antibiotics, autogenous *S. suis* vaccines have become very popular in Europe.

**Main body:**

Autogenous vaccines (AV) are generally neither tested for safety, immunogenicity nor protective efficacy, which leads to substantial uncertainties regarding control of disease and return on investment. Here, *S. suis* publications are reviewed that include important data on epidemiology, pathologies and bacterin vaccination relevant for the use of AV in the field. Differences between herds such as the porcine reproductive and respiratory syndrome virus infection status and the impact of specific *S. suis* pathotypes are probably highly relevant for the outcome of immunoprophylaxis using autogenous *S. suis* bacterins. Thus, a profound diagnosis of the herd status is crucial for management of expectations and successful implementation of AV as a tool to control *S. suis* disease. Induction of opsonizing antibodies is an in vitro correlate of protective immunity elicited by *S. suis* bacterins. However, opsonophagocytosis assays are difficult to include in the portfolio of diagnostic services.

**Conclusion:**

Autogenous *S. suis* bacterins are associated with limitations and risks of failure, which can partly be managed through improvement of diagnostics.

## Background

Worldwide, numerous research groups work on the identification of protective antigens of *Streptococcus* (*S.*) *suis*. Different proteins of *S. suis* have been expressed as recombinant antigens in *Escherichia coli* and used after purification as vaccination antigens in challenge trials with mice or pigs (for review see [1]). Hopefully, these scientific efforts result in the development and licensing of a cross-protective *S. suis* vaccine, as *S. suis* diseases constitute a major burden for pig producers, not only regarding return on investment but also due to animal welfare and the use of antibiotics. Though in some countries *S. suis* vaccines are available, there is currently no *S. suis* vaccine approved by the European Medicines Agency (EMA). As the pressure for reduction of the use of antibiotics in veterinary medicine increases in Europe, autogenous *S. suis* vaccines have become a main tool in porcine practice. However, it is hoped that licensed vaccines will become available to combat *S. suis* disease burden. This review is based on the current scientific *S. suis* literature and the authors’ experience with diagnostic services, experimental *S. suis* infections and many years of consultation of practitioners with the objective to improve the use of autogenous *S. suis* vaccines.

### The epidemiology of *S. suis* diseases in Europe

*S. suis* is a very diverse pathogen. Currently 29 confirmed serotypes are described for this species [2]. Worldwide, *S. suis* serotype 2 is most frequently isolated from clinical cases of *S. suis* disease [3]. Wisselink et al. [4] serotyped 411 *S. suis* strains from 7 European countries isolated from diseased pigs between 1991 and 1997. Thirty-two percent (*n* = 132) and 20% (*n* = 84) of the strains belonged to serotypes 2 and 9, respectively. However, serotype 9 has become the most prevalent serotype among invasive isolates in some European countries with a large pig industry such as the Netherlands and Spain [3, 4]. Prüfer et al. [5] compared *S. suis* strains isolated between 1996 and 2004 and 2015–2016 in Germany and recorded a reduction of *cps*2 strains (including not only serotype 2 but also 1/2, see below) among invasive isolates from 44.3 to 23.5%, respectively. The authors speculate that the increased use of autogenous vaccines (AV) in recent years led to this reduction. Furthermore, reduction of serotype 2 cases through AV may have paved the way for serotype 9 emergence as discussed by Büttner et al. [6]. Serotypes 1 (and 14), 4 and 7 are also important serotypes contributing to invasive diseases in Europe [3–5, 7].

Very different strains may belong to the same serotype. This is well known for serotype 2 harboring highly virulent, moderately virulent and strains considered avirulent based on the results of epidemiological studies and experimental infections [8–10]. Recent research has also highlighted the diversity of serotype 9 in Chinese *S. suis* isolates [11]. Furthermore, a collection of serotype 9 isolates in the Netherlands was shown to contain some strains with the ability to cause severe disease and others only inducing mild clinical signs which was confirmed in experimental infection of piglets [12]. Virulent serotype 2 strains in Europe generally carry the genes *epf*, *mrp* and *sly* encoding the extracellular factor, the muramidase-released protein and the hemolysin suilysin, respectively [8, 9, 13].

Various laboratories have used multilocus sequence typing (MLST) to compare *S. suis* strains and a large online database is available which allows to determine the sequence type (ST) of a *S. suis* isolate after sequencing 7 housekeeping genes (https://pubmlst.org/ssuis) [14]. Closely related sequence types may form a clonal complex (CC) indicating evolutionary expansion. Strains of CC1 and CC16/87 are responsible for more than 40% of invasive *S. suis* diseases in Europe [3]. Whereas CC1 harbors mainly serotype 2 strains, serotype 9 dominates in CC16/87 [3, 14]. Based on data of experimental infections, strains of CC16 are considered less virulent than strains of CC1 [15]. However, many well-managed herds in Europe experience severe problems with this pathotype and AV in these herds are often unsuccessful (personal communication between practitioners and authors).

There are very important differences regarding the prevalence of *S. suis* genotypes among geographical regions. Important examples are the high prevalence of virulent *mrp*+ *epf*+ *sly*+ *cps2* strains of CC1 and of mrp* *cps9* of CC16/CC87 in Europe in contrast to the situation in North America, where *S. suis cps2* strains of CC25 and CC28 are dominating among invasive isolates [3, 10]. However, recent data from the United States revealed a shift in the prevalence of CCs during the past years with CC25 no longer being a dominant CC, whereas prevalence of the diverse CC94 has increased [16]. In the Netherlands, numerous cases of severe disease were associated with serotype 2 strains of ST20 [17]. Furthermore, 40 recently sequenced Spanish serotype 9 strains belonged to CC61 [18], which is distantly related to CC16/CC87 [19]. We recently described emergence of invasive diseases due to serotype 7 (*cps7*) strains of ST29 in Germany [20]. Thus, the MLST database and publications on the prevalence of *S. suis* serotypes and sequence types suggest that there are also important differences between European countries, though piglets are frequently transported from one country to another. Regarding the complex epidemiology of *S. suis* diseases, the number of sequence-typed strains in Europe is still rather low. A systematic unbiased typing of invasive isolates from various European countries has not been conducted to date. For a more comprehensive review of the prevalence and geographical distribution of *S. suis* sequence types/CCs we refer the reader to Goyette-Dejardins et al. [3].

Interestingly, it was recently shown that there are differences in virulence among *cps*2 strains of CC28 [21]. This implies that in certain cases *cps* typing and MLST might not be sufficient to estimate the virulence potential of a strain which adds to the complexity of profiling biologically relevant *S. suis* genotypes. However, in many cases, determination of the serotype and the ST is sufficient to identify a virulent *S. suis* pathotype [16].

Although *S. suis* isolates are genotyped by diagnostic services for practitioners on a large scale in various European countries such as Germany and Austria, investigations on putative differences in the prevalence of *S. suis* genotypes between herds and different pig lineages within a country, have not been published to the best of our knowledge. This is however highly relevant for herd management and the use of AV.

### Proper sampling and reasoning for genotyping of *S. suis* isolates prior to generation of AV

In case of clinical signs that raise the suspicion of a *S. suis* herd problem, thorough clinical examinations of different affected animals and proper sampling of sites of infection during necropsies of sacrificed animals is highly recommended. In most piglets with central nervous system disorders due to *S. suis* meningitis bacteriological investigations of brain swabs and/or cerebrospinal fluid will generate unambiguous results as long as the piglet was not treated with antibiotics prior to sampling. Appropriate samples in case of a septicemic animal are internal organs such as spleen and liver. Legislature restricts necropsies on farms in some European countries. In Germany for example, the animal by-products disposal law (§10) generally prohibits the skinning, opening and cutting of animal carcasses on farms. Thus, if necropsies are not an option, cerebrospinal fluid (CSF) and heparinized blood are in our experience excellent samples for bacteriological investigations of animals with a suspected diagnosis of *S. suis* meningitis and septicemia, respectively. Sufficient anesthesia is the least requirement for a CSF aspirate. However, for animal welfare reasons we recommend sacrificing piglets with central nervous system disorders for sampling of CSF. To avoid transporting the carcasses long distances to the nearest pathological examination facility, these samples may be obtained from animals at the respective farm. For detection of *S. suis* bacteremia heparinized blood might be directly streaked on Columbia blood agar plates. This is a convenient approach to screen numerous piglets without clinical signs of disease but elevated body temperature for *S. suis* bacteremia. Although it is not in accordance with the classical microbiological blood culture, we have successfully used this method over many years to obtain invasive *S. suis* isolates from live animals.

Piglets that have been found dead might also be investigated but care should be taken with regard to post-mortem contamination, especially of the liver and peritoneal swabs. Alpha hemolytic streptococci including *S. suis* are very often among the contaminating bacteria. Contaminations might also easily occur in the case of swabs taken from the bicuspid and tricuspid valves. The heart should be opened with a new sterile pair of scissors to prevent these contaminations. In case of arthritis, removal of hair and subsequent thorough sterilization of the skin or even removal of the skin should be conducted prior to sampling of the joints. Isolates of such samples can generally be regarded as invasive as these sites are sterile in healthy animals. However, this is not the case for isolates obtained from samples of the respiratory tract as *S. suis* colonizes the upper respiratory tract of piglets physiologically in high numbers. *S. suis* isolates from lung samples collected in necropsies conducted hours after an animal’s death are not necessarily related to the disease of the animal. Including these isolates in an AV should be carefully evaluated.

**Fig. 1:**
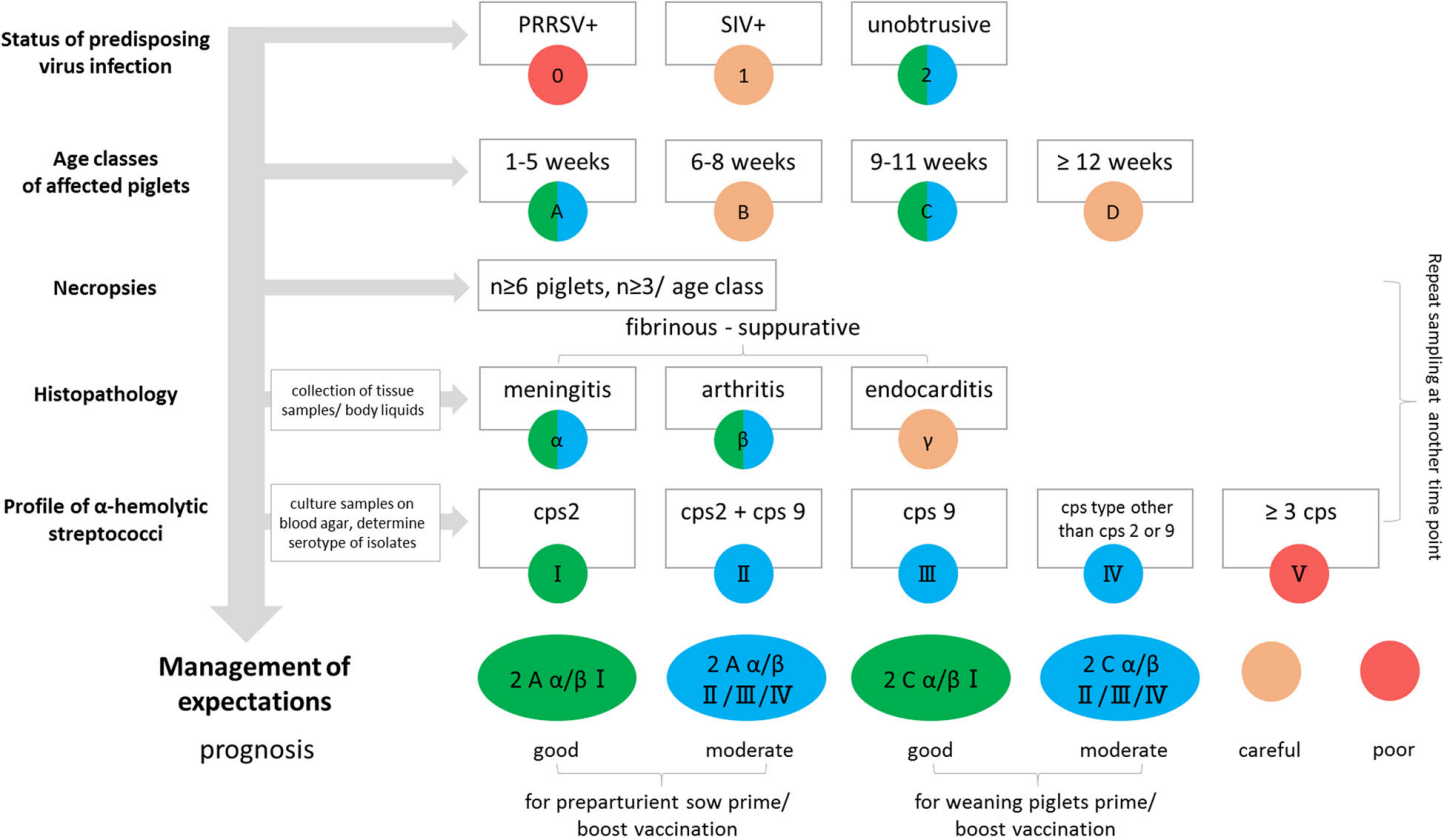
Management of expectations for the implementation of *S. suis* autogenous bacterins in preparturient sow or weaning piglet prime-boost vaccination based on a profound herd diagnosis including virus infections, histopathological investigations and profiling of *S. suis* pathotypes. For management of expectations we propose that it is crucial to determine the status of virus infection in the herd, the age classes of affected piglets, the localization and character of lesions in pathological investigations and the genotype(s) of the invasive *S. suis* strains. Herds with PRRSV infection and/or *S. suis* diseases associated with three or more different *cps* types have in our opinion a poor prognosis with regard to the protective effectiveness of the AV. SIV infection should lead to a careful prognosis. For 6 to 8-week-old piglets, it is generally difficult to find a working vaccination protocol as maternal immunity does not lead to protection in 8-week-old piglets and priming in suckling piglets is regarded as ineffective [22]. Since endocarditis is associated with biofilm formation and bacteria in biofilms are not killed by opsonizing antibodies, the prognosis in herds with piglets showing endocarditis should also be careful. *S. suis* diseases undergo a dynamic process in affected herds. Thus, we generally recommend to repeat herd diagnostics at least once prior to generating an autogenous bacterin (at least the bacteriological investigations including genotyping of the *S. suis* isolates should be repeated)

Based on our experiences we think it is important to investigate numerous piglets to verify the suspected cause of the herd problem and to obtain invasive isolates from different animals. It is difficult to give a justified recommendation for the number of piglets to be investigated. However, we are aware of herd problems associated with more than four different *S. suis* genotypes and of *S. suis* infections of herds occurring intermingled with *Glaesserella (G.) parasuis* (*Haemophilus parasuis*) infections. In our opinion the number of investigated animals should not be less than 4 (better 6) and investigations should be repeated at an additional time point if feasible (Fig. 1).

One could argue that an AV should include all invasive *S. suis* isolates obtained from different animals and that typing of isolates does not provide reliable information on the protective efficacy of the AV. However, typing of isolates might be useful (i) to give a justified claim on the role of predisposing factors in this herd as isolation of multiple different genotypes indicates strong predisposition (see below), (ii) to manage expectations as protective efficacies of *cps*9 bacterins are generally lower than of *cps*2 bacterins (see below), (iii) to detect new infections in the herd related to *S. suis* patho−/genotypes not included in an AV generated in the past and (iv) to relate one’s own experience with specific compositions of autogenous bacterins.

### Profiling of *S. suis* pathotypes in herd management

For the reasons mentioned above, typing of virulence-associated genes of *S. suis* isolates via multiplex (MP) PCRs has become a very important diagnostic service in Europe and is offered by numerous laboratories using different protocols [2, 7, 23]. Nowadays virtually all large swine practices in North Western Germany use these services or even conduct genotyping themselves. Genotyping by MP-PCR was first introduced by Peter Valentin-Weigand’s group at the Institute for Microbiology of the University of Veterinary Medicine Hannover (Germany) covering the capsule biosynthesis operons *cps*1, *cps*2, *cps*7 and *cps*9 as well as the virulence markers *mrp*, *epf* and *sly* [7]. Detection of *gdh* and *arc*A were included to confirm the putative diagnosis of *S. suis* [24, 25]*.* MP-PCRs used for profiling of *S. suis* isolates do not allow reliable detection of large variants of *epf* [7, 23]. Thus, an additional *epf* monoplex PCR should be conducted to identify strains with variants of the *epf* genes (designated epf*) encoding extracellular factors (EF*) larger than the 110 kDa EF [7, 26]. Differentiation of size variants of *mrp* and *epf* through additional PCRs might also be used for fine typing of *S. suis* strains belonging to *cps*1, *cps*2, *cps*7 and *cps*9 [7, 20, 27, 28]. Identification of *mrp*+ epf* *sly*+ *cps*2+ is biologically relevant as this pathotype is considered to be moderately virulent in comparison to highly virulent *mrp*+ *epf*+ *sly*+ *cps*2 and experimentally avirulent *mrp*- *epf*- *sly*- *cps*2 strains in Europe [8, 9, 26]. Although many *S. suis* studies have been published since this original work by Vecht et al. [9], the results of this comparison of 12 wt strains in experimental infection of germfree pigs remains significant for differentiation of pathotypes within serotype 2 strains in Europe [9].

The genes *mrp*, *epf* and *sly* have been used for numerous years by diagnostic services in Central Europe as virulence markers. However, there are other virulence markers that might be used as well. Differentiation of putative pilus gene clusters as described by Takamatsu et al. [29] is a further approach to identify virulent strains, as *srtBCD* plus *srtF* and *srtF* plus *srtG* genotypes are associated with virulent strains of CC1 and CC27, respectively. Furthermore, other genes such as the gene encoding surface antigen one (SAO) harbor repetitive sequences and show size variations in *S. suis* which might be used for PCR-based typing of *S. suis* isolates similar to *mrp* and *epf* typing [30, 31] .

Differentiation of *cps* operons via PCR is generally in accordance with serotyping, but important deviations are known. As pointed out in various previous publications [3, 7, 23] serotypes 2 and 1/2 as well as serotypes 1 and 14 cannot be distinguished using any of the published PCR protocols. This implies putative consequences for the composition of AV. If diseases in a herd were due not only to serotype 2 but also to serotype 1/2 infections, this would be misdiagnosed as a herd problem related only to one serotype (*cps*2) using diagnostic services based on virulence-associated gene profiling. As a consequence, only one serotype might be included in the AV. To overcome this restriction, differentiation of serotype 2 and 1/2 via serotyping or *cps*K sequencing is recommended [3, 32]. Whether this is of importance in the case of these closely related serotypes is, however, not known. In the last years further MP-PCR assays were described finally covering all known serotypes [2]. Of note, *S. suis* research has focused very much on serotype 2 and has to some extent covered also serotypes 9, 7 and 1. Information on other serotypes except for data on their prevalence is not available.

Any of the described MP-PCRs detecting *cps*1, *cps*2, *cps*7 and *cps*9 as well as the virulence-associated factors *mrp*, *epf* and *sly* allow identification of important pathotypes such as *mrp*+ *epf*+ *sly*+ *cps*2 and *mrp*+ *sly*+ *cps9* strains. However, the informative value for strains not belonging to any of the four *cps* types is rather low, even more as *mrp* and *epf* are only confirmed virulence markers within *cps*2 [8]. Accordingly, a very low positive predictive value was found testing *mrp*, *epf* and *sly* for all *S. suis* strains of an out-of-sample collection [33]. Recently, Wileman et al. [33] conducted a genome-wide association study to identify new genetic markers for invasive diseases and asymptomatic carriage. The authors introduced a novel MP-PCR which putatively allows differentiation of these two groups of *S. suis* strains through detection of three genes (SSUST30534, SSU1589 and SSU0207) which have so far to the best of our knowledge not been used for profiling of *S. suis* isolates within diagnostic services. The gene SSUST30534 encoding a putative ATP sugar transporter is associated with asymptomatic carriage. So far, this tool developed by Wileman et al. [33] has been tested to the best of our knowledge only for *S. suis* strains isolated in Great Britain. We imagine that this novel MP-PCR approach could also become a valuable tool for diagnostic services, especially for the screening of sows during quarantine. However, as it does not differentiate important pathotypes such as mrp* *cps*9 strains of CC16 and *mrp*+ *epf*+ *cps*2 strains of CC1, the application of this new method for the characterization of invasive isolates used for composition of an AV is less convincing.

### Virus infections putatively disturbing the protective efficacy of a *S. suis* bacterin

Virus infections predispose piglets for invasive *S. suis* diseases. This has been shown experimentally for porcine reproductive and respiratory syndrome virus type 2 (PRRSV) by different groups [34–36]. As an example, the vast majority of piglets (20 of 22) born from gilts experimentally infected with PRRSV were prone to a lethal course of disease elicited by intranasal *S. suis* serotype 2 infection in contrast to piglets from non-infected gilts (only 5 of 23 with severe disease) [34]. In addition to experimental data, outbreaks of PRRSV infections in Southeast Asia were associated with an increase in *S. suis* diseases [35, 37, 38]. In 2006, an unprecedented epidemic of porcine high fever syndrome (PHFS) associated with high mortality was recorded in China. This outbreak was caused by infections with highly pathogenic PRRSV with gene deletions in NSP2 and GP5 and secondary infections mainly caused by *S. suis* serotype 7. The devastating outcome of this coinfection was reproduced experimentally [35]. *S. suis* serotype 7 is generally considered less virulent than serotype 2. Accordingly, intranasal infections with serotype 7 alone did either result in mild [35] or no clinical signs of disease (own unpublished data). Although one should be careful not to directly extrapolate experimental results obtained with PRRSV type 2 to PRRSV type 1, main immunomodulatory mechanisms between these two virus infections are similar and therefore analogies in clinical consequences during bacterial co-infections might be present to a large extent [39].

We have recorded great differences among herds in the number of *S. suis* genotypes causing disease at a time (unpublished results). As there are substantial differences in virulence among *S. suis* strains, we claim that these differences between herds are related to predisposing factors such as PRRSV infection. If PRRSV is circulating in a herd, *S. suis* strains being only weakly virulent by themselves will get the chance to cause disease in contrast to herds which are not subject to strong predisposition. Therefore, the heterogeneity of *S. suis* strains isolated from internal organs of diseased pigs in a herd seems to be related to the impact of main predisposing factors such as PRRSV infection.

We have been investigating numerous diseased pigs over many years from two PRRSV free herds and have with one exception detected only a single *S. suis* pathotype from diseased pigs in both herds, namely a *mrp*+ epf* *cps*2 strain of CC 1 and a ST94 *mrp*+ *sly*+ *cps*9 strain though other genotypes are frequently detected on the mucosal surfaces and the tonsils. In herds that experience *S. suis* diseases only in association with a single highly virulent *S. suis* pathotype, although numerous other *S. suis* serotypes and STs are present on the mucosal surfaces, predisposing virus infections may be less important for this herd problem. The clinical outcome is very much determined by the virulence of the *S. suis* strain. This has been shown in comparative experimental infections [9, 15, 21]. In any case, PRRSV infection is likely to increase the severity of the clinical outcome.

For veterinary practice, it is important to ask if the protective efficacy of bacterins is also influenced by predisposing virus infections. However, very few researchers have addressed this question. Halbur et al. [40] compared different antibiotic treatments and autogenous bacterin vaccination to control experimental *S. suis* infections secondary to infection with the highly virulent VR-2385 PRRSV strain. Vaccination of PRRSV infected piglets with a *S. suis* serotype 2 bacterin failed to elicit significant protection against a homologous challenge. Ceftiofur treatment was the only regime leading to significantly reduced mortality. The study by Halbur et al. [40] lacks a group not preinfected with PRRSV. Thus, it is not entirely clear if the experimental PRRSV infection really deteriorated the protective efficacy of the serotype 2 bacterin. This seems, however, reasonable, as vaccination with a serotype 2 bacterin including the same adjuvant, Emulsigen, elicited significant protection against a homologous challenge in a different study [41]. Although more research on the interference of PRRSV infection with vaccination against *S. suis* is needed, it seems overall very likely that PRRS caused by an immune modulating virus infection has negative effects on the protective efficacies of *S. suis* bacterins.

Coinfections of swine influenza virus and *S. suis* have also been investigated by different groups [42–45]. Experimental coinfection of pigs with influenza H1N1 virus and *S. suis* serotype 2 leads to increased mortality compared to single influenza H1N1 virus and *S. suis* serotype 2 infection [44]. Coinfection outbreaks of influenza and *S. suis* have also been reported in the field in England [46]. Accordingly, different in vitro studies have shown that swine influenza H1N1 and H3N2 infections promote the ability of *S. suis* serotype 2 to adhere to and invade respiratory epithelial cells [42, 43, 45]. Whether the protective efficacy of a *S. suis* bacterin is influenced by influenza virus infections occurring either during vaccination or concomitantly with *S. suis* infection has to the best of our knowledge not been addressed in any study. However, we speculate that this might be the case e. g. through the exacerbated expression of cytokines such as CCL2, CCL4, IL-6, IL-8, and TNFα [42].

There are certainly more virus infections such as Porcine Circovirus type-2 (PCV2) with a putative influential effect on *S. suis* infection and vaccination, but their role in the epidemiology of *S. suis* diseases remains to be determined.

### Experimental data on protective efficacies and immunogenicities of *S. suis* bacterins

No commercial vaccine is available for protection against disease caused by various *S. suis* pathotypes [1]. Until today, pigs in Central Europe can only be vaccinated by AV. Invasive isolates obtained from usually sterile tissues of diseased pigs of a specific herd should be used to prepare an AV (bacterin) for this herd.

*S. suis* serotype 2 bacterins have been shown to elicit protection against homologous challenge by different groups [22, 41, 47, 48]. However, this does not mean that every autogenous serotype 2 vaccine is protective. Wisselink et al. [48] compared *S. suis* serotype 2 bacterins with a water-in-oil and aluminium hydroxide adjuvant. The authors observed protection against mortality only with the water-in-oil adjuvant and not with aluminium hydroxide. Furthermore, the vaccination protocol is also very important. Prime-boost vaccination in the 2nd and 4th week of life with an autogenous *S. suis* serotype 2 bacterin failed to induce seroconversion. Accordingly, significant protection was not observed in these early vaccinated piglets [22]. In contrast, prime-boost vaccination after weaning with a serotype 2 vaccine containing the same adjuvant resulted in significant protection against mortality [41]. Based on these results we think that inhibition by maternal antibodies is very relevant in the field and do not recommend *S. suis* bacterin vaccination of suckling piglets (at least not priming prior 3 weeks of age). This is in agreement with the advice by Haesebrouck et al. [49] not to immunize piglets before an age of 3–4 weeks. The authors highlight the importance of an adequate vaccination scheme which should include prime-boost vaccination with an interval of at least 2 weeks and a gap of 2 weeks between boost and the time of risk. It is important to note that priming with a *S. suis* serotype 2 bacterin is generally not sufficient to elicit protection as was shown in a challenge experiment conducted 2 weeks after bacterin priming in the 6th week of life [50]. A putative explanation for the lack of protection during the early adaptive immune response elicited by serotype 2 bacterin priming is that *S. suis*, in contrast to other bacterial pathogens, expresses an IgM-specific protease [51]. Thus, protection might only occur after induction of high specific IgG titers including opsonizing IgG obtained by booster vaccination. However, we showed for serotype 7 (*cps*7) strains of CC29 that IgM plays an important role in restricting survival of this pathotype in porcine blood. As IgM levels increased after weaning in the course of the early natural adaptive immune response, this pathotype was efficiently killed in porcine blood by IgM [20]. Virulent *S. suis* strains express numerous virulence factors contributing to survival in porcine blood as reviewed in depth by Fittipaldi et al. [52]. In our opinion, one should be careful to generalize conclusions obtained for a single strain/pathotype to the entire *S. suis* population as there are great differences among strains.

In the field, AV are very often made of different *S. suis* strains, e. g. *S. suis* serotypes 2 and 9, or even as combinations with other pathogens such as *G. parasuis*. We are not aware of any publication describing the immunogenicity and protective efficacy of a multivalent *S. suis* bacterin. Therefore, any combination goes along with the risk of interfering with the protective efficacy of the serotype 2 bacterin. If the vast majority of diseases in growing piglets are caused by serotype 2, we recommend application of a monovalent *S. suis* bacterin with an appropriate adjuvant in the 4th and 6th week, as this has been demonstrated to lead to protection. However, there are cases which call for variations, e.g. if two serotypes are of comparable importance for disease in a herd. Furthermore, *S. suis* diseases between the 6th and 8th week of life make it difficult to give a justified recommendation: Preparturient vaccination of sows with a serotype 2 bacterin covers the 6th but not the 8th week of life [22] and prime-boost vaccination of piglets in the 2nd and 4th week of life is not a solution to this problem in our opinion (see above). A vaccination regimen with vaccination of sows prior to farrowing and prime-boost vaccination of piglets in the 4th and 6th of life did not result in protection against an intravenous homologous challenge in the 8th week of life [22]. We are not aware of data showing how vaccination of suckling or weaning piglets might be successfully combined with preparturient sow vaccination or how the aforementioned gap in protection can be closed. In conclusion, a detailed herd analysis with valid data on peaks of *S. suis* diseases is important to manage expectations prior to introduction of an AV (see Fig. 1).

A *S. suis* serotype 2 bacterin prime-boost vaccination after weaning led to significant protection against a homologous challenge but failed to elicit significant protection against a heterologous serotype 9 challenge [41]. In the field it is also generally accepted that bacterins elicit at their best homologous but not heterologous protection (personal communication). Opsonizing antibodies induced by prime-boost serotype 2 bacterin immunization of weaning piglets lead to killing of the homologous serotype 2 strain but not a heterologous serotype 9 strain [6]. The level of opsonizing antibodies is determined in an in vitro assay measuring bacterial survival in the presence of purified granulocytes, the pathogen and serum to be investigated. In this assay opsonizing antibodies bind to the surface of the pathogen and induce directly or indirectly (e.g. through complement activation) opsonophagocytosis and finally killing of the bacteria in phagolysosomes of the neutrophils. Antibody-mediated engulfment of *S. suis* by granulocytes induces generation of reactive oxygen species which finally leads to killing of this pathogen [53]. In contrast to ELISAs this assay measures functionality of antibodies. Importantly, levels of opsonizing antibodies were found to correlate with the protection elicited by bacterin vaccination [41]. Piglets with high titers of opsonizing antibodies survived the challenge until the end of the experiment in contrast to piglets with low levels of opsonizing antibodies. However, opsonophagocytosis assays are currently to the best of our knowledge not offered as a diagnostic service in Central Europe. Whether such tests might be useful to optimize AV immunization, should be subject of further investigations. The purification of porcine neutrophils requires availability of fresh blood and makes the assay in comparison to ELISAs cumbersome and difficult to integrate into the portfolio of a diagnostic laboratory.

IgM and IgG antibodies directed against the capsular polysaccharides can induce opsonophagocytosis [54]. Since polysaccharides are T cell independent antigens, vaccination with free capsular polysaccharides does not induce IgG antibodies. In contrast, vaccination with serotype 2 capsule polysaccharides conjugated to tetanus toxoid elicits prominent capsule specific IgM and IgG titers [54]. Opsonizing antibodies induced by serotype 2 bacterin prime-boost vaccination can be absorbed with an unencapsulated mutant [41]. This observation indicates that at least a substantial fraction of the opsonizing antibodies elicited by serotype 2 bacterins is not directed against the capsule polysaccharides. Piglets undergo diverse immune responses upon bacterin vaccination. We have detected antibodies opsonizing serotype 9 after vaccination with a serotype 2 bacterin in individual animals (unpublished data). These observations suggest that *S. suis* serotype 2 expresses cross-protective antigens in vitro but that these antigens are not the main immunogens of a bacterin.

Very limited data is available on protective efficacies of *S. suis* serotype 9 bacterins though this serotype is more important than serotype 2 in some European countries [3, 4]. Prime-boost vaccination in the 4th and 6th week of life elicited protection against mortality caused by experimental intravenous challenge with a serotype 9 strain of CC16 [6], which includes most of the invasive serotype 9 strains isolated in Europe [3]. Importantly, vaccinated piglets surviving the challenge showed signs of disease namely fibrinous endocarditis [6]. This is an important observation as active endocarditis might lead to serious complications including meningitis even in piglets with high titers of antibodies mediating efficient killing of planktonic bacteria [55]. However, as bacteria are covered with fibrin in the vegetation on the heart valves, they are to a large extent protected against killing by opsonizing antibodies. *S. suis* serotype 9 was shown to efficiently form biofilms in vitro [56] and in vivo [6, 55]. Furthermore, levels of opsonizing antibodies elicited through serotype 9 bacterin vaccination against the homologous serotype 9 strain were rather low (Table 1). Thus, we propose that immunoprophylaxis by bacterins against serotype 9 is more limited than serotype 2 bacterins.

**Table 1:** Immunogenicities and protective efficacies of *S. suis* serotypes 2 and 9 bacterins after prime-boost vaccination of weaning piglets and homologous challenge experiments using the same oil-in-water adjuvant (Emulsigen) [6, 41]

Genotype of the bacterin and the challenge strain	*mrp* + *epf* + *sly* + *cps*2-		mrp* *sly* + *cps*9	
	bacterin	placebo	bacterin	placebo
MRP-ELISA^a,b^	500 Units	5 Units	100 Units	25 Units
Mean bacterial survival factor in opsonophagocytosis assays^b,c^	0.4	1.6	0.78	1.18
Mortality	28.6%	87.5%	22.2%	77.8%
Morbidity	28.6%	100%	77.8%	100%
Pathohistological score^d^ of infected piglets	1.25	3.75	2.8	3.8

^a^Using rMRP of serotype 2 strain 10 as antigen

^b^These assays were conducted with samples collected 2 weeks after the boost vaccination and prior to the experimental infection

^c^Survival factors were determined by dividing the number of colony forming units (CFUs) after a 2 h incubation period at 37 °C by the number of CFUs at t = 0

^d^For calculation of the pathohistological score the sum of the highest scores of each animal for any of the investigated organs was divided by the number of animals (ω = Σscore_max_/*n*_animals_)

Dekker et al. [57] conducted a comprehensive study designed to determine the impact of serotype 9 bacterin vaccination on colonization and transmission, rather than on protection against mortality. The homologous bacterin failed to prevent colonization and transmission of *S. suis* serotype 9. Based on these results it is clear, that practitioners should not expect to reduce the pressure of infection in a herd by implementation of a serotype 9 AV. Whether serotype 2 bacterins lead to reduction of transmission and colonization of serotype 2 is not clear. In a different study, the serotype 2 challenge strain was isolated from the tonsils of bacterin-vaccinated piglets 2 weeks after challenge suggesting that vaccinated piglets will not eliminate the challenge strain completely from the mucosal surfaces [41]. Based on the results of these experimental studies and observations in the field, we doubt that bacterin vaccination is effective enough to build up herds free of a specific *S. suis* pathotype. However, it has been suggested that the prevalence of serotype 2 infections decreased in Europe due to the use of autogenous serotype 2 bacterins [5]. Furthermore, Swildens et al. [58] describe combined amoxicillin treatment and bacterin vaccination of preparturient sows as a successful method to eliminate *S. suis* serotype 2 from the tonsils of the carrier sows and to prevent respective colonization of their piglets. However, it remains to be demonstrated whether this is a reliable tool to build up herds free of a specific *S. suis* pathotype.

Data on the immunogenicity and protective efficacies of *S. suis* bacterins containing other serotypes than 2 and 9 is even scarcer. Results of a field study on the use of an autogenous serotype 7 vaccine did not reveal clear protection against disease, at least not in the case of vaccination in the first and third week of life [27]. Passive maternal immunity induced by a serotype 14 bacterin was shown to only partially protect piglets against clinical signs of disease [59].

It is important to remember that there are many variables in the generation of an AV which are crucial for the immunogenicity and protective efficacy. As mentioned above, it has been shown for *S. suis* bacterins that the choice of the adjuvant is critical [48]. A systematic comparison of numerous adjuvants has unfortunately not been published. Though results of *cps*2 bacterins with water-in-oil adjuvants are encouraging with regard to protective efficacies, they are generally associated with severe side effects such as high body temperature and anorexia for numerous hours. In addition to the adjuvant, conditions selected for inactivation of the bacteria may have a great influence on the immunogenicity and the protective efficacy. Important epitopes may be lost through harsh inactivation conditions. Formaldehyde concentrations used for inactivation of *S. suis* in vaccination trials varied substantially. Dekker et al. [57] used 0.5% formaldehyde for 15 min to inactivate *S. suis cps*9 at room temperature. In contrast, Wisselink et al. [48] inactivated *cps*2 with 0.3% formaldehyde at 4 °C overnight, and Peter Valentin-Weigand’s group used 0.1% formaldehyde overnight at 8 °C for *cps*2 and *cps*9 [6, 22, 41]. However, a concentration of 0.1% formaldehyde is the borderline value for complete killing of *S. suis* leading sometimes to survival of single bacteria.

In conclusion and as recently evaluated in a field study [60], the use of AV is generally reasonable in serotype 2 infected herds. Both sow and weaning piglet vaccination might be successful. Regarding other serotypes the efficacy of immunization with an autogenous bacterin is questionable. The vaccination scheme and adjuvant used are important influencing factors for the immunogenicity and efficacy of a bacterin. Finally, an important disadvantage of AV is the lack of information on vaccine safety and efficacy data compared to commercially produced vaccines [49].

### Legal basis for the use of AV in Europe

The European Union Directive 2001/82/EC defines AV (autogenous products) as „Inactivated or non-inactivated immunological veterinary medicinal products which are manufactured from pathogens and antigens obtained from an animal or animals from a holding and used for the treatment of that animal or the animals of that holding in the same locality”(Article 3).The use and production of AV is regulated at the national level in the European Member States as the Directive 2001/82/EC excludes them from legal standards and does not define criteria for their quality, safety, efficacy and potency [61]. Therefore, legislation for AV differs between the member states, but has many similarities [62]. For the first time, the new Regulation (EU) 2019/6 on veterinary medicinal products of the European Parliament and of the Council of December 2018 sets requirements for the production and quality control of AV (https://eur-lex.europa.eu/legal-content/EN/TXT/PDF/?uri=CELEX:32019R0006&from=EN). Regulation 2019/6 defines AV as “inactivated immunological veterinary medicinal products which are manufactured from pathogens and antigens obtained from an animal or animals in an epidemiological unit and used for the treatment of that animal or those animals in the same epidemiological unit or for the treatment of an animal or animals in a unit having a confirmed epidemiological link” (Article 2(3)). Articles 94 (Good manufacturing practice, GMP), 105 (Veterinary prescriptions), 108 (Record-keeping by owners and keepers of food-producing animals), 117 (Collection and disposal of waste of veterinary medicinal products), 120 (Advertising prohibition for AV), 123 (Controls by competent authorities) and 134 (Prohibiting the supply of veterinary medicinal products) of the new regulation shall apply to AV. The regulation 2019/6 will lead to the harmonization of standards for production and quality control within Europe. Therefore, it will be necessary to set up detailed GMP guidelines to ensure the quality of AV in the future. Meanwhile the European Manufacturers of Autogenous Vaccines and Sera (EMAV) was set up as an association of AV producers to „ensure high level product quality standards based on requirements determined by laws, regulations and technical standards and standards set out by the association “(https://www.emav.be). However, as the regulation 2019/6 will enter into force on 28th January 2022 the existing requirements for production and use of AV still differ between EU Member States [61–63].

## Conclusion

Understanding the epidemiology of *S. suis* diseases is crucial for using bacterins successfully. Furthermore, a comprehensive herd screening covering main porcine viruses as well as differentiation of *S. suis* pathotypes is important for managing expectations on autogenous bacterins. Although different groups have demonstrated protection of *S. suis* serotype 2 bacterins against experimental homologous challenge, the protective effectiveness of AV used in the field is to the best of our knowledge not documented. The authors propose that a database including diagnostic data collected prior and post introduction of autogenous bacterins as well as protocols used for generation of bacterins and vaccination strategies would increase the scientific foundation substantially.

## Data Availability

Data sharing is not applicable to this article as no datasets were generated or analyzed during the current study.
